# *QuickStats*: Percentage* of Adults Aged ≥18 Years Who Received Care at Home from a Friend or Family Member During the Past 12 Months,^†^ by Age Group — National Health Interview Survey,^§^ United States, 2021

**DOI:** 10.15585/mmwr.mm7223a8

**Published:** 2023-06-09

**Figure:**
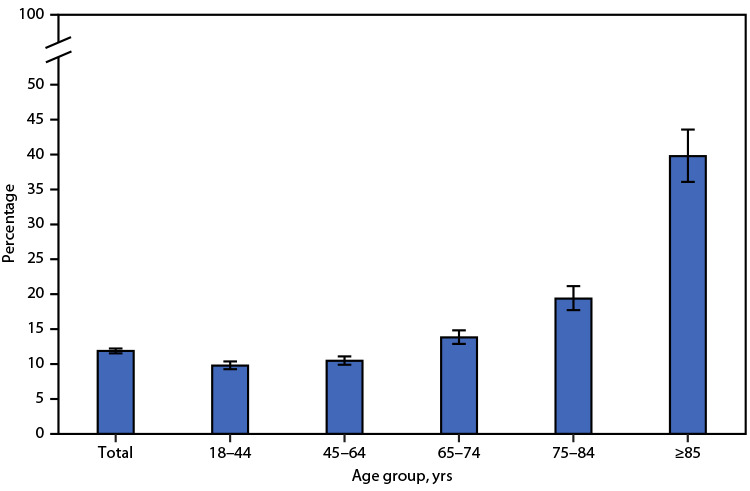
During 2021, 11.9% of adults aged ≥18 years received care at home from a friend or family member during the past 12 months. The percentage of adults who received care during the past 12 months was similar among adults aged 18–44 years (9.8%) and 45–64 years (10.5%), then increased with age to 13.8% among those aged 65–74 years, 19.4% among those aged 75–84 years, and more than doubled to 39.8% among those aged ≥85 years.

**Source:** National Center for Health Statistics, National Health Interview Survey, 2021. https://www.cdc.gov/nchs/nhis/index.htm

